# Potential for Front of Pack Labeling Exposure to Impact US Dietary Choices: A Population-Based Cross-Sectional Study Using NHANES 2017–2018

**DOI:** 10.3390/nu14142995

**Published:** 2022-07-21

**Authors:** Elizabeth K. Roark, Colin D. Rehm, Christina L. Sherry

**Affiliations:** 1 PepsiCo R&D, Plano, TX 75024, USA; 2 PepsiCo R&D, Purchase, NY 10577, USA; colin.rehm@pepsico.com (C.D.R.); christina.sherry@pepsico.com (C.L.S.)

**Keywords:** food labeling, front-of-pack labeling, food packaging, diet surveys, cross-sectional studies, NHANES, United States, health status, diet behaviors

## Abstract

In recent years, front-of-pack nutrition labeling (FOPL) schemes have proliferated, but the components of the diet subject to FOPL have not been described. This study quantified the proportion and elements of the diet that would be subject to FOPL in the US. The 2017–2018 National Health and Nutrition Examination Survey (*n* = 7121; age ≥2 year) 24-h dietary recalls were used to identify foods/beverages subject to FOPL. The proportion of dietary energy and additional dietary constituents subject to FOPL was estimated. Overall, 57% of dietary energy would be subject to FOPL. Individuals consuming more away-from-home meals had lower exposure to FOPL. Adults with a healthy-weight and those consuming a more healthful diet had more exposure to FOPL. Protein, sodium, potassium, whole fruit, vegetables, and unprocessed meats were less subject to FOPL as compared to total sugars, added sugars, calcium, fruit juice, milk, yogurt, nuts/seeds and whole grains. Because less than 60% of the diet would be impacted by FOPL, implementation of such a policy may have limited reach for the US diet and demonstrates some inconsistencies with current dietary guidance regarding the under- and over-representation of key food groups and nutrients.

## 1. Introduction

Nutrition labeling is commonly mandated to appear on food packages, and in the United States (US), this takes the form of the Nutrition Facts Panel (NFP). In 2016, the NFP was updated, with the changes implemented by 2020. All packaged foods regulated by the Food and Drug Administration must provide information on the serving size, number of servings, total energy, and selected macro- and micronutrients. Manufacturers can choose to provide information for other micronutrients. The nutrients mandated for inclusion in the NFP were selected based on their relationship to chronic disease risk or evidence that under/over-consumption may be of public health concern [1]. The evidence is mixed for whether the NFP helps consumers make healthy choices [2,3,4,5]. Between 30–61% of adults report using NFPs all or most of the time when purchasing groceries and typically only devote a few seconds to reviewing this information [4,6,7].

An emerging global strategy with the goal of improving population-wide dietary intakes is the use of front of package labeling (FOPL). These graphical labels and/or images are intended to provide a quick overview of the nutritional quality of a product, complementing the more detailed NFP. The use of FOPL started as early as 1989 in Sweden; however, since 2011 there has been an introduction of at least 13 FOPL systems [8]. Although traditionally voluntary, FOPL has now been implemented in over 30 countries and 10 are mandatory [9]. Underlying a FOPL scheme is a nutrient profiling (NP) model that classifies or ranks foods according to their nutritional composition with the implicit goal of promoting a healthful diet through guided choice [10]. NP models are heterogeneous in terms of dietary constituents included and reference sizes leading to between-system differences. Some FOPLs focus only on dietary constituents to limit (e.g., sodium or saturated fat) and others include both constituents to limit and to encourage (e.g., fiber, vegetables). Some systems apply to all food categories equally, while some are modified for specific food groups (e.g., dairy or beverages). Systems can also vary in terms of whether they utilize scoring (i.e., assigns a summary indicator reflective of overall nutritional value, such as Nutri-Score or Health Star Rating [HSR]) or a threshold (i.e., nutrient limits to qualify/disqualify, such as “Stop Signs”) [11].

Although there has been an increase in the number and use of FOPL systems, there is limited research on their real-world impact. Studies have focused on the ability of the FOPLs to discriminate the nutritional quality of food products [12,13], purchase intent [14], food purchasing [13], and perceived healthfulness [14,15]. However, a fundamental question has gone unanswered. Analyses quantifying the proportion of the diet that would be subject to FOPL are lacking. This information is critical in assessing the extent by which FOPL can impact dietary choices, as well as identifying which population sub-groups and dietary constituents would be most or least impacted. To fill these gaps, we used 2017–2018 National Health and Nutrition Examination Survey (NHANES) data to demonstrate how FOPL could impact the US diet and support health promotion.

## 2. Materials and Methods

### 2.1. Data Source & Population

The 2017–2018 NHANES, a population-based nationally-representative survey of the dietary intakes and health status of the non-institutionalized US population was used for the present study. All data are publicly available on the Centers for Disease Control and Prevention and United States Department of Agriculture websites [16,17]. Ethics approval was obtained by the National Center for Health Statistics and informed consent was obtained for all participants [18]. Individuals age ≥2 year (excluding breastmilk consumers) completing a valid 24-h recall were included (*n* = 7121). Individuals who reported being pregnant or breastfeeding were included in all analyses, so the data are representative of the total US population. A complete case analysis approach was used in all analyses. There were no additional inclusion/exclusion criteria.

### 2.2. 24-h Recall Methods

The in-person 24-h dietary recall conducted in the NHANES Mobile Examination Center (MEC) by trained staff, was the primary source of dietary data. The 24-h recall records all foods and beverages consumed in the previous 24-h. The computer-assisted recall starts with an initial overview of the previous days eating occasions, with subsequent passes gathering more detailed information [19]. The recall was done by a parent/guardian for children age ≤11 (child could assist 6–11 year) [19]. A single 24-h recall provides an unbiased estimate of population-level average intakes, which was the primary metric of interest [20].

### 2.3. Estimating Dietary Exposure to Foods/Beverages Eligible for FOPL

A multi-step approach was developed to identify foods that would be eligible for FOPL with the goal of determining the population-level exposure to FOPL for numerous dietary constituents (e.g., dietary energy, vegetables). Because many foods consumed by NHANES participants may be multi-ingredient items, that include a mix of foods eligible or not eligible for FOPL (e.g., for a hamburger the bun and sauces may be eligible, but the ground beef and fresh vegetables may not be eligible), the underlying ingredients table within the 2017–2018 Food and Nutrient Database for Dietary Studies (FNDDS) was used to determine whether each ingredient was eligible. Given the popularity of Nutri-Score, the Nutri-Score technical guidance was used to determine whether a product was eligible for FOPL [21]. This approach is generally consistent with the presence/absence of the NFP on the food item. Excluded foods include fresh produce, fresh unprocessed meat, eggs, plain water, herbs/spices, salt, sugar substitutes, non-ready-to-drink coffee/tea, chewing gums, baby foods and infant formula.

The FNDDS ingredients database query (*n* = 2402) was done independently by the authors (one author coded the entire database, and another coded a sub-sample of 200 items, over-sampling frequently consumed items). In analyses weighted for the frequency of consumption, the agreement was 93.9% and Kappa, which corrects for random agreement, was 0.88, indicating a high-level of agreement. Systematic discrepancies in coding were discussed and amendments to the coding strategy were made. Additional foods described in the database as being “frozen”, “canned”, “ready-to-eat”, “ready-to-heat” were flagged as being eligible for FOPL. All foods and beverages obtained from the following sources were determined to not be eligible for FOPL: fast food/full-service restaurants, cafeterias, grown/caught, sport/recreation facility, or street vendors.

Using the FNDDS ingredients table allowed for the merging of data on nutrient and food group content for each ingredient from FNDDS for nutrients and the Food Patterns Ingredients Database (FPID) for food groups. Retention factors and moisture adjustments were accounted for and the total contribution of FOPL eligible foods to each dietary constituent (e.g., calories, vitamin D, whole grains) was estimated. These data were then summed for each participant to allow for population-level estimation of exposure to each dietary constituent from FOPL vs. non-FOPL eligible sources. Further stratifications were done to separate the data into three groups: FOPL, not FOPL but purchased at stores, and away-from home (AFH) foods not eligible for FOPL. Dietary constituents evaluated included energy (kcal), total fat, saturated fat, monounsaturated fat, polyunsaturated fat, solid fat (a USDA composite measure that combines naturally occurring fats solid at room temperature and hydrogenated and partially hydrogenated fats), protein, carbohydrate, total sugar, and added sugars. Fiber, potassium, vitamin D, calcium, and iron were also assessed as they were identified in recent DGA as being nutrients of public health concern either for the total population or for specific sub-groups (e.g., iron) [1] and are included in the NFP. Food pattern equivalents (e.g., cup or ounce-equivalents) were obtained for whole fruit, fruit juice, dark-green vegetables, red/orange vegetables, potatoes, other starchy vegetables, milk, cheese, yogurt, unprocessed red meat, processed meat, poultry, seafood, organ meat, legumes, nuts/seeds, soy, eggs, refined grains and whole grains. Food group values were obtained from the 2017–2018 FPID and Food Patterns Equivalents Database (FPED) [16].

### 2.4. Covariates

We also examined how the dietary exposure to FOPL differed by population socio-demographics, including age group (2–9; 10–19; 20–34; 35–49; 50–64; 65 year+), gender, race/ethnicity (non-Hispanic white, non-Hispanic Black, Hispanic, non-Hispanic Asian/Pacific Islander, other race/mixed race), family income-to-poverty ratio (<1.0, 1–1.99, 2–3.99, ≥4.0), education among adults ≥25 year (<HS, HS, some college, ≥college graduate), marital status (married/living with partner, widowed, divorced/separated, never married), and family size (1, 2, 3, 4, 5, ≥6). The poverty measure is the ratio of family income to the federal poverty guidelines; in 2018, this value was $16,240 for a family of 2 [22].

Potential differences in dietary exposure to FOPL were also assessed by diet behaviors, diet quality and health behaviors. Diet-related behaviors assessed included being the primary food shopper, number of AFH meals in the past week (≤1, 2, 3, 4–5, ≥6), and number of ready-to-eat foods purchased from grocery stores (quartiles). Diet quality was measured by the Healthy Eating Index-2015 (HEI-2015) [23,24]. HEI-2015 analyses were conducted for the total population and stratified by age (children/adolescents vs. adults) due to underlying differences in HEI-2015 values (i.e., children having higher HEI values) [1]. Other health behaviors assessed included average weekly recreational physical activity quantified as moderate-equivalent minutes per-week in quartiles [25]. Smoking status was grouped into current, former and never. The number of dietary supplements used in the last 30 days was grouped into quartiles. Differences in dietary exposure to FOPL by health status including body weight as measured by body mass index (BMI), weight loss intent in the prior year, self-reported health status, diagnosed diabetes, hypertension, high-blood pressure and cardiovascular disease (defined as heart attack, coronary heart disease, angina or stroke) was also assessed. Besides BMI, all health variables were based on self-report and analyses of health-related variables were limited to adults ≥20 year.

### 2.5. Analysis Approach

To determine population-level dietary exposure to FOPL by socio-demographics, diet/health behaviors, and health outcomes the proportion of dietary energy subject to FOPL was estimated. In addition to crude analyses, age-adjusted analyses were conducted using the 2000 US Standard Population [26]. Age-adjustment was done as age was a strong predictor of exposure to FOPL. Wald tests for survey-weighted data were used to determine if there were statistically significant differences in the population ratio in pairwise comparisons. Trends for quantitative ordinal variables (e.g., age or family income) were tested by including the covariate in a survey-weighted Poisson regression model. A similar approach was used to estimate the proportion of each dietary constituent of interest (e.g., total fat, fruit, or sodium), however, age-adjustment was not used as the primary interest was in the overall population effect. The proportion of each dietary constituent subject to FOPL was compared to the proportion for total energy using a Wald test. Analyses accounted for the complex survey design of NHANES, were weighted to represent the United States population in 2017–2018 and were conducted in Stata 16.1 (College Station, TX, USA).

## 3. Results

### 3.1. Population Characteristics and Potential Exposure to FOPL

**Figure 1:**
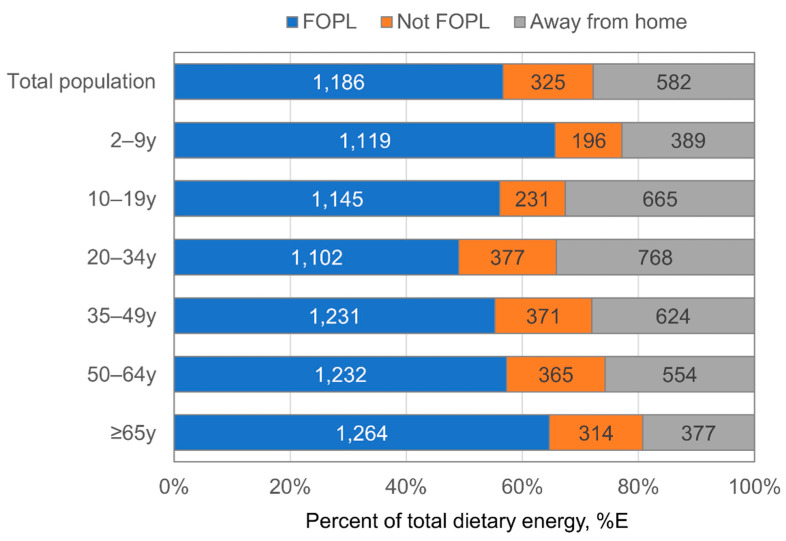
Daily energy (kcal/d) subject to FOPL (front of package labeling), not subject to FOPL but obtained from a store or other source where FOPL would be implemented and away from home among US population age ≥2 year, 2017–2018.

Table 1 shows the characteristics of the 7121 participants by age, gender, race/ethnicity, education, family-income to poverty ratio and other socio-demographic factors. Because NHANES is a nationally-representative survey the sample characteristics are representative of the US population in 2017–2018. Figure 1 shows the average number of calories that would be subject to FOPL, would not be subject to FOPL (but purchased in a store), or from AFH sources and therefore not subject to FOPL. Overall, 56.6% of energy (%E) or 1186 kcals/d would be subject to FOPL. AFH foods/beverages accounted for 27.8%E (583 kcals/d) while foods purchased in stores and not subject to FOPL accounted for 15.5%E (325 kcals/d). The proportion of dietary energy subject to FOPL varied by age in a U-shaped manner by age, with children 2–9 year (65.7%E) and older adults ≥65 year (64.6%E) having the most exposure to FOPL as compared to 20–34 year, who had the lowest exposure (49.1%E). This pattern was explained by differences in AFH foods, which varied nearly 2-fold comparing older adults (19.3%E) versus those 20–34 year (34.2%E). The proportion of energy not subject to FOPL but purchased at stores was consistent across age among adults (~16.1–16.8%E) and children (~11.3–11.5%E).

Also shown in Table 1 is the average %E that would be subject to FOPL overall and by key subgroups. FOPL exposure did not differ by gender. No significant differences in FOPL were observed by race/ethnicity. In both crude and age-adjusted analyses, a negative trend was observed for exposure to FOPL and family income, with individuals from lower-income families having greater exposure to FOPL than higher-income individuals. For education, those with less than a high-school education had higher exposure to FOPL. Widowed and divorced/separated adults had greater exposure to FOPL than adults who were married/living with a partner in both crude and age-adjusted analyses. Family size was not associated with major differences in exposure to FOPL.

### 3.2. Diet & Health Behaviors & Differences in FOPL Exposure

Table 2 shows the average %E subject to FOPL classified by diet behaviors, diet quality and health behaviors. A strong association between AFH meals and FOPL was observed, with individuals consuming more AFH meals having considerably less exposure to FOPL. For HEI-2015 quintiles, adults ≥20 years with the highest HEI scores had the highest exposure (61.5%E), resulting in an additional ~134 kcals/day eligible for FOPL vs. the lowest quintile. While all other HEI quintiles had >50%E exposure to FOPL, no clear trend was observed across these categories. Additional analyses found that AFH meals explained the association between HEI and the proportion of energy subject to FOPL among adults (*p*-trend = 0.017 in age-adjusted analyses and *p*-trend = 0.22 after adjustment for AFH foods). No association between HEI and %E subject to FOPL was observed for children. In age-adjusted analyses no difference was observed in the proportion of the diet subject to FOPL by number of grocery ready-to-eat foods consumed in the past 30 days, physical activity, smoking or supplement use.

### 3.3. Health Status & FOPL Exposure

Table 3 shows the average %E subject to FOPL by weight status and prevalent conditions. Adults who were overweight or obese had somewhat lower exposure to FOPL than healthy weight individuals. Relatedly, those who stated they had tried to lose weight in the last year had less exposure, resulting in approximately 75 less kcal/day from FOPL eligible foods. A U-shaped relationship was observed between self-reported health status and potential exposure to FOPL; individuals with excellent or fair/poor health having the most exposure, while those reporting very good health had the least exposure. Having a diagnosis of diabetes or cardiovascular disease also resulted in higher exposure, but this association was no longer present after age-adjustment. Diagnosed hypertension and high cholesterol were not associated with exposure to FOPL.

### 3.4. Diet Exposed vs. Not Exposed to FOPL

**Figure 2:**
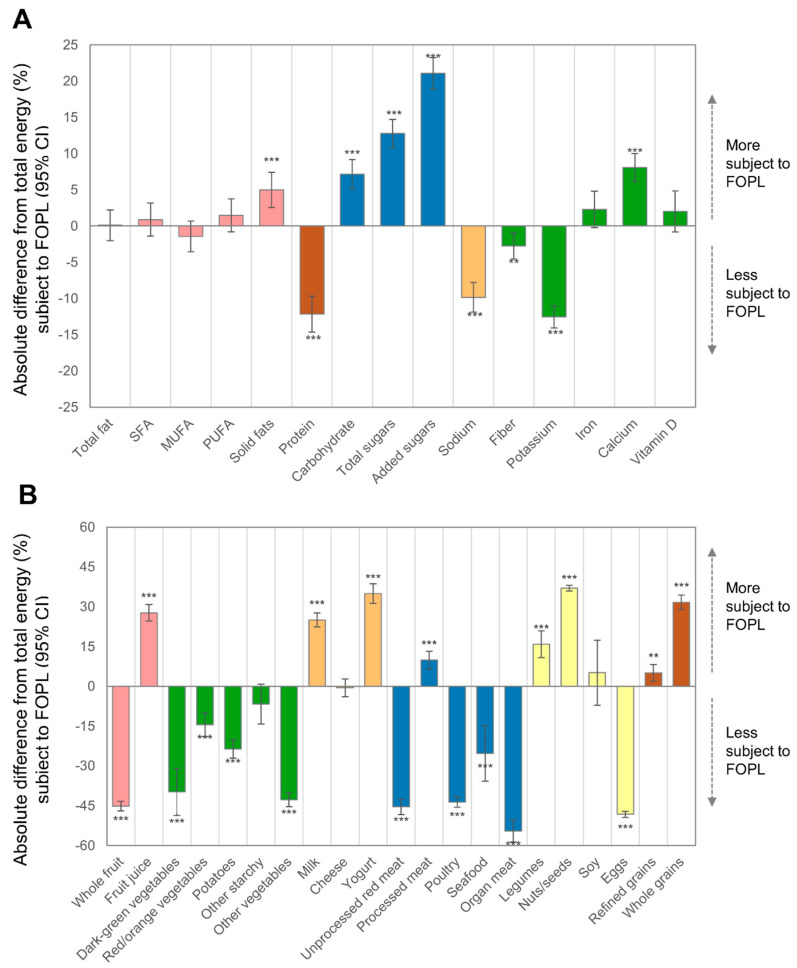
Absolute differences in proportion of macronutrients and micronutrients (**A**) and food groups (**B**) subject to FOPL within the diet of US population age ≥2 year (*n* = 7121), 2017–2018. Asterisks indicate *p*-value comparing absolute difference to total energy where 56.6% is subject to FOPL; *** *p* < 0.001; ** 0.001 < *p* < 0.01. For example, 44.5% of protein consumed is subject to FOPL so the absolute difference compared to energy (56.6%) is −12.2% (95% CI −14.7, −9.7, *p* < 0.001). SFA stands for saturated fatty acids, MUFA stands for monounsaturated fatty acids and PUFA stands for polyunsaturated fatty acids.

Figure 2A and Table 4 shows the proportion of macronutrients and micronutrients subject to FOPLs. For macronutrients, protein was under-represented as compared to energy, whereas, total carbohydrate, total sugar and added sugars were over-represented. No difference was observed for individual fatty acids, except for solid fat, which was more subject to FOPL. Sodium, fiber and potassium were under-represented, while calcium was over-represented. No difference was observed for iron or vitamin D.

Food group intake is shown in Figure 2B and Table 4. Approximately 1/3 and 1/4 of fruit and vegetable intakes, respectively, were subject to FOPL. Fruit eligibility was heterogeneous comparing fruit juice (85% eligible) to whole fruit (11.5%). For vegetables, the other starchy vegetables sub-category had the highest proportion eligible with half of intake being eligible. All other vegetable sub-categories were under-represented, especially other vegetables (13.9%) and dark green vegetables (16.9%). Compared to other food groups, milk (81.7%) and yogurt (91.5%) were over-represented; however, the proportion of cheese subject to FOPL did not differ from the total contribution of FOPL foods compared to total energy. Overall, one-quarter of meat, poultry, seafood and protein foods were eligible for FOPL. Processed meats (66.5%) were over-represented, whereas unprocessed red meat, poultry, organ meat and eggs were all less than 15%. Most nuts/seeds (93.6%), legumes (72.5%), and to a lesser extent soy (61.8%), were eligible for FOPL. Marked heterogeneity was also observed for grains, with ~90% of whole grains being eligible for FOPL compared to ~62% of refined grains. The different results for grain sub-types were mostly attributable to the large contribution of AFH foods to refined grains, but not whole grains.

## 4. Discussion

Nutrition labelling, including FOPL, has been identified as one strategy to address growing global concern regarding sub-optimal diet [27]. With approximately 43% of dietary energy at the population-level not subject to FOPL, our analysis additionally identifies opportunities (e.g., consideration of nutrients and/or food groups that align with dietary guidelines, creative and more effective AFH nutrition labeling practices, FOPL schemes that work for all SES groups) to better leverage the display of nutrition information. For no sub-population analyzed in terms of socio-demographics, dietary/health behaviors or health status, did FOPL exposure exceed 66% of total dietary energy. With the notable exceptions of age and number of AFH meals, differences in the proportion of dietary energy subject to FOPL by socio-demographics, dietary behaviors and health status were modest indicating that most population sub-groups would have comparable exposure to FOPL. Some dietary constituents would be impacted by FOPL to a greater or lesser extent, suggesting that if implemented the effects on dietary intakes and health may be uneven.

### 4.1. SES and Food Label Use

Our data showed that individuals with less education and lower incomes had modestly higher potential exposure to FOPL than individuals of higher socioeconomic status. Separate research reveals that these populations tend to have a lower level of understanding of traditional nutrition labels and use them less often [6,28]. With ~60% FOPL exposure among vulnerable populations, an opportunity exists to improve diet quality by drawing attention to easy-to-comprehend food labeling elements that may promote a higher quality diet. Some FOPL schemes (e.g., scoring-type with summary indicator) are better understood across socioeconomic status levels [29,30] and should be further explored to ensure they grab consumer attention. Some public health interventions have been shown to inadvertently widen health disparities due to higher-SES individuals being earlier or more ardent adopters [31]. While the real-world impact of FOPL implementation on disparities in dietary intakes in the US is uncertain, based on our observation that lower SES individuals have marginally higher exposure to FOPL it is unlikely that such an intervention would significantly widen disparities. On the other hand, we did observe that adults with the highest quality diets in terms of HEI-2015 had the greatest exposure to FOPL, suggesting that based on current dietary habits those in a position to benefit least from FOPL currently have the greatest exposure. Additional analyses adjusting for AFH meals explained the association between diet quality (HEI-2015) and FOPL exposure, highlighting the importance of AFH foods in any link between FOPL exposure and diet quality.

### 4.2. Away from Home Food Sources

In this analysis, just over half of daily energy came from foods and/or beverages subject to FOPL with the remaining energy coming from AFH sources or store-based purchases of foods not subject to FOPL. The influence of FOPL on overall diet quality is therefore potentially limited given that a third of daily energy comes from AFH sources. The impact of AFH foods on FOPL exposure was strongest for younger adults (20–34 year) who had a 2-fold higher %E from AFH sources and ~15% less exposure to FOPL as compared to older adults and young children. While family income and race/ethnicity are both associated with AFH food consumption the key driver behind food AFH consumption was age [32].

Although nutritional quality can vary between AFH establishments, when compared to meals prepared at home, AFH meals contain higher levels of saturated fat, sodium, and refined grains, and lower levels of nutrients to encourage such as calcium and fiber [32], and have been associated with larger portion sizes and higher per meal calorie content [33]. As such, more frequent AFH consumption tends to be associated with poor diet quality [32,34]. Further, our analysis identified lower FOPL exposure for people with overweight/obesity, which may be explained by higher AFH consumption [35,36]. In the post-recessionary period, AFH consumption has remained stable and accounts for a substantial proportion of dietary intakes [32]. A nutrition labeling strategy that includes AFH food sources, in addition to FOPL, is likely to have a greater impact on diet quality. The US has had mandatory restaurant menu labeling since 2018 but the regulation applies to chain restaurants with ≥20 locations and requires that only calories be displayed. Additional written nutrition information (e.g., saturated fat, sodium, sugars, fiber and protein) are available by request [37]. Food groups and other nutrients to encourage are not included. Although research on the effectiveness of current AFH labeling is mixed [38], further research exploring novel and creative ways (e.g., contextual, interpretative, digital, etc.) of presenting nutrition information at point of consumption, is warranted and ongoing [38].

### 4.3. Nutrient/Food Group Intake & FOPL Exposure

Nutrient profile models that underpin FOPLs should ideally complement current government advice on a healthy diet [10]. It is therefore important that nutrients and food groups to both limit and encourage are considered in FOPL schemes. Our data reveals major differences between these key nutrients and those eligible for FOPL leading to potential inconsistencies between FOPL and dietary guidance. The DGA recommends limiting consumption of sodium, saturated fat and added sugars. Consistent with prior work showing added sugars preferentially come from stores, more than three-quarters of added sugars is subject to FOPL. Saturated fat is well covered (57.5%) by FOPL. However, less than half of sodium intake is subject to FOPL. Although sodium reduction efforts for packaged foods should continue, public health impact would likely be greater if reductions extended beyond just packaged foods. There are two key sources not subject to FOPL: AFH foods and table salt used during cooking or eating. Those ≥20 year reporting eating at least one meal at a restaurant consumed 53% of their daily sodium intake from AFH food sources, resulting in an additional 605 mg/day compared to those not consuming restaurant food [39].

The DGA identified potassium, calcium, vitamin D, and dietary fiber as being “shortfall” nutrients in the US diet. In our analysis, 40–50% of potassium and fiber intakes are subject to FOPL. Fresh fruits and vegetables, which are exempt from nutrition labeling, are key sources of both nutrients and explain their under-representation. Our data show that >60% of calcium intake is subject to FOPL and is driven by the FOPL exposure of dairy, especially milk and yogurt. While just over half of cheese intake is subject to FOPL, AFH pizza consumption is a key contributor to cheese intake which explains the difference between cheese and other forms of dairy [40]. Since calcium is a mandatory nutrient for nutrition labeling in US, including calcium or dairy content in FOPL could help draw attention to food sources delivering this shortfall nutrient. At present, most NP/FOPL schemes do not include dairy or calcium, but some scores do use alternative thresholds for dairy foods (e.g., HSR in Australia and New Zealand). Americans also fail to meet the recommendations for key food groups, including fruit and vegetables (80–90% shortfall), dairy (90% shortfall) and whole grains (98% shortfall) [1]. Apart from fruit juice, fruit and vegetable intakes were less subject to FOPL. Vegetables and fruit are both important components of some FOPL algorithms, including Nutri-Score and HSR, but a potential discrepancy exists at the population level for these food groups, as a very low proportion of their intake is subject to FOPL. Innovative ways to conveniently incorporate fruits and vegetables into FOPL eligible foods and beverages may be one way to address the fruit and vegetable intake challenge. Further, inclusion of meaningful fruit and vegetable quantities into FOPL algorithms would further draw consumer attention to FOPL eligible fruit and vegetables sources including frozen and canned fruits and vegetables. Education on the nutrient content of frozen and canned fruits and vegetables, specifically how they are comparable to fresh may also help Americans meet recommended intakes. Additionally, Americans are recommended to increase the variety of consumption of protein subgroups (seafood, beans, peas and lentils) and our analysis found that while less than 50% of protein intake is subject to FOPL, plant-based sources of protein, legumes and nuts/seeds, are more subject to FOPL, warranting a discussion as to whether these identified foods groups to encourage should be included in FOPL schemes.

Nearly 90% of whole grain intake is potentially subject to FOPL; however, whole grains are not typically included in the NP models that underpin the FOPL [41,42]. While fiber is a purported surrogate for whole grains, our analysis shows fiber and whole grains intakes at the population-level to have differing proportions subject to FOPL indicating that fiber may not be an appropriate surrogate for whole grain content. This finding is consistent with prior research examining the HSR system which showed that fiber was not an adequate proxy for whole grain content [42]. Whole grains are generally an excellent source of fiber, but the fiber content of one full serving (16 g) of whole grains can vary from about 1 to 3 g. While some whole grains deliver less fiber, they still provide other valuable nutrients (e.g., antioxidants, iron, B vitamins, etc.). Fiber alone is, therefore, not enough to assess whether a product is in fact a whole grain food [43]. Future FOPL schemes may want to carefully consider including whole grains, particularly considering that sub-optimal whole grain intake is the second leading dietary cause of Disability Adjusted Life Years according to the Global Burden of Disease study [44].

### 4.4. Strengths and Limitations

Numerous study limitations should be noted. First, the proportion of the diet subject to FOPL was estimated indirectly as data was not always collected on the form in which foods were consumed. Furthermore, we used Nutri-Score criteria for including specific foods, which may differ from other systems. These issues may lead to random or systematic measurement error in our estimation. Food categories potentially more subject to misclassification include processed meats and mixed dishes. Furthermore, data were based on self-reported dietary intakes which may also be subject to random and systematic measurement error via imprecision in food descriptions, portion sizes and/or omitted foods. Lastly, this study is not able to determine whether individuals will use the information provided in a FOPL, indicating that our assessment of 57% of the diet being subject to FOPL is likely an over-estimate of the amount of the diet at the population-level that could be influenced by FOPL. Strengths of the study include the fact that it was population-based and used a robust and flexible dietary assessment instrument (e.g., this study would not be possible using food frequency questionnaire data). The use of actual dietary intake data as opposed to sales/purchase data is an additional strength, as sales-based data could not be used to directly estimate the proportion of the diet or dietary constituents subject to FOPL.

## 5. Conclusions

The use of FOPL as a population-based intervention to address sub-optimal diet has been widely proposed and implemented in recent years. With this first-ever quantification of the proportion of the diet and dietary components that would be more/less subject to FOPL, several key factors have been uncovered in how the implementation of a FOPL could impact the US diet. Except for age and frequency of AFH food consumption, FOPL would impact population sub-groups defined by demographics, health behaviors and health status uniformly. The public health impact of FOPL can be augmented by other labeling tools that address the contribution from AFH foods. Lastly, a FOPL system and the underlying NP model should align with the DGA and complement key public health messages. Our results demonstrate this is not always the case given the under-representation of some food groups and nutrients. Taken together, these data indicate that when aligned with current dietary guidance and in combination with other communication tools, FOPL could be a useful public health tool.

## Figures and Tables

**Table 1:** Population characteristics and proportion of daily dietary energy impacted by FOPL (front of package labeling) in the United States, 2017–2018.

			Mean Percent %E Impacted by FOPL (95% CI)	
	*n*	%	Crude	Age-adjusted
Total	7121	100.0	56.6 (54.6, 58.7)	56.8 (55.0, 58.7)
Age group, y				
2–9	1053	10.3	65.7 (62.8, 68.6) ***	-
10–19	1327	13.5	56.1 (52.4, 59.8) **	-
20–34 [ref]	1079	21.1	49.1 (45.9, 52.2)	-
35–49	1057	18.4	55.3 (52.0, 58.6) **	-
50–64	1384	20.9	57.3 (54.6, 59.9) ***	-
≥65	1221	15.7	64.6 (62.2, 67.1) ***	-
*p*-trend			0.012	
Gender				
Female [ref]	3640	51.2	57.5 (55.5, 59.5)	57.4 (55.4, 59.5)
Male	3481	48.8	56.0 (53.4, 58.6)	56.4 (54.0, 58.8)
Race/ethnicity				
Non-Hispanic white [ref]	2491	59.4	57.6 (54.4, 60.8)	57.4 (54.2, 60.6)
Non-Hispanic Black	1659	11.7	54.0 (52.1, 56.0)	54.7 (52.9, 56.4)
Hispanic	1633	17.9	54.4 (51.6, 57.1)	55.6 (53.2, 58.0)
Non-Hispanic Asian/Pacific Islander	862	5.6	56.1 (52.9, 59.3)	56.7 (53.1, 60.2)
Other race/mixed race	476	5.5	59.8 (54.9, 64.6)	60.4 (56.8, 64)
Family income-to-poverty ratio				
<1.00 (lower income)	1419	16.4	58.6 (54.5, 62.8)	59.5 (56.5, 62.5) *
1–1.99	1794	21.7	59.3 (56.6, 62.1) *	59.4 (56.6, 62.2) **
2–3.99	1662	27.3	57.4 (53.6, 61.2)	57.7 (54.4, 61.0) *
≥4.00 (higher income) [ref]	1459	34.6	53.6 (50.9, 56.4)	53.9 (51.6, 56.2)
Missing	787	-	-	-
*p*-trend			0.019	0.003
Education (among adults ≥25 year)				
<HS	840	11.1	61.5 (58.2, 64.9) *	60.1 (56.8, 63.4) *
HS	1005	26.0	57.7 (53.3, 62.1)	55.7 (51.9, 59.6)
Some college	1396	30.8	54.9 (52.1, 57.7)	54.6 (52.1, 57.2)
≥College [ref]	1086	32.1	56.3 (53.2, 59.4)	55.4 (52, 58.8)
*p*-trend			0.057	0.054
Marital status (adults)				
Married/living with partner [ref]	2805	61.8	55.5 (53.3, 57.8)	55.0 (52.8, 57.1)
Widowed	363	6.0	61.6 (58.0, 65.3) **	63.2 (58, 68.4) **
Divorced/separated	717	12.8	61.8 (57.9, 65.8) **	60.6 (56.7, 64.5) *
Never married	852	19.5	51.3 (47.0, 55.5)	57.3 (54, 60.6)
Family size				
1	1050	15.5	54.2 (50.5, 57.9)	54.9 (51.3, 58.5)
2	1463	24.2	57.6 (54.7, 60.5)	54.7 (51.2, 58.2)
3	1183	17.3	56.2 (53.4, 59.1)	57.3 (54.4, 60.1)
4 [ref]	1353	17.5	55.7 (51.7, 59.7)	56.4 (52.2, 60.5)
5	1020	13.8	57.9 (52.7, 63.2)	58.4 (53.7, 63.2)
≥6	1079	11.7	58.8 (54.5, 63.1)	59.4 (55.1, 63.7)
*p*-trend			0.22	0.069

Asterisks indicate results of pairwise test comparing each proportion to the specified reference group identified as [ref] above: *** *p*-value < 0.001; ** 0.001 < *p*-value < 0.01; * 0.01 < *p*-value < 0.05. Age-adjusted analyses not conducted for age group.

**Table 2:** Proportion of population’s diet in terms of daily energy impacted by FOPL in the United States by diet behaviors, diet quality and selected health behaviors, 2017–2018.

			Mean Percent %E Impacted by FOPL (95% CI)	
	*n*	%	Crude	Age-adjusted
Primary food shopper (age ≥20 year)				
No	1776	38.7	54.0 (50.6, 57.4)	54.1 (51.1, 57.0)
Yes [ref]	2965	61.1	57.1 (55.2, 59.0)	56.7 (54.8, 58.5)
AFH meals in past week				
≤1 [ref]	2804	33.7	65.1 (63.0, 67.2)	64.2 (62.2, 66.3)
2	1321	19.0	60.9 (57.4, 64.4) *	60.9 (57.8, 64) *
3	987	14.1	54.4 (50.7, 58.2) ***	54.1 (50.6, 57.6) ***
4–5	1005	15.5	51.8 (48.2, 55.4) ***	53.4 (50.3, 56.5) ***
≥6	973	17.7	45.5 (42.9, 48.0) ***	46.8 (44.6, 49) ***
*p*-trend			<0.001	<0.001
Grocery ready-to-eat foods in past 30 days				
None [ref]	4580	59.8	57.2 (54.8, 59.6)	57.1 (55.0, 59.3)
T1: 1–2	1189	18.5	58.1 (56.0, 60.3)	58.0 (56.0, 60.2)
T2: 3–5	706	11.1	55.7 (51.7, 59.7)	55.8 (52.2, 59.3)
T3: ≥6	609	10.6	52.4 (47.9, 57.0)	53.8 (50.3, 57.3)
*p*-trend			0.16	0.16
Quintiles of Healthy Eating Index-2015 (total population)				
Q1: 6.3–36.8	1468	19.9	56.0 (52.7, 59.4) **	56.9 (53.6, 60.1)
Q2: 36.8–44.9	1473	20.2	53.6 (49.9, 57.3) ***	54.5 (51.2, 57.8) ***
Q3: 44.9–52.3	1390	20.0	56.5 (54.0, 59.1) **	57.1 (54.8, 59.4) **
Q4: 52.3–61.3	1362	20.0	55.6 (51.4, 59.7) *	55.3 (51.3, 59.3) *
Q5: 61.3–97.9 [ref]	1428	19.9	61.9 (60.0, 63.9)	61.0 (58.6, 63.3)
*p*-trend			0.002	0.028
Quintiles of Healthy Eating Index-2015 (adults ≥20 year)				
Q1: 6.3–36.8	880	18.6	55.0 (51.3, 58.7) **	55.2 (51.1, 59.3) **
Q2: 36.8–44.9	942	20.3	52.0 (47.4, 56.6) ***	52.4 (48.2, 56.6) ***
Q3: 44.9–52.3	927	20.0	55.5 (52.5, 58.6) ***	55.9 (53.1, 58.7) **
Q4: 52.3–61.3	933	20.2	54.2 (49.2, 59.2) **	53.3 (48.4, 58.1) **
Q5: 61.3–97.9 [ref]	1059	20.9	62.2 (60.2, 64.2)	61.5 (59.7, 63.3)
*p*-trend			0.001	0.017
Quintiles of Healthy Eating Index-2015 (children 2–19 year)				
Q1: 6.3–36.8	588	24.3	58.7 (53.7, 63.8)	-
Q2: 36.8–44.9	531	19.9	59.3 (56.1, 62.6)	-
Q3: 44.9–52.3	463	19.8	60.4 (55.7, 65.1)	-
Q4: 52.3–61.3	429	19.4	61.0 (56.2, 65.8)	-
Q5: 61.3–97.9 [ref]	369	16.6	60.4 (53.9, 66.8)	-
*p*-trend			0.43	
Moderate-equivalent recreational physical activity per week (adults ≥20 year)				
None [ref]	2529	45.9	56.9 (54.6, 59.1)	55.6 (53.6, 57.7)
T1: 10–180 min	876	21.0	56.9 (53.6, 60.2)	56.8 (53.6, 60.0)
T2: 190–450 min	631	15.2	53.2 (49.6, 56.9) *	54.0 (50.3, 57.7)
T3: ≥455 min	699	18.0	54.1 (50.0, 58.1)	56.8 (53.0, 60.5)
*p*-trend			0.035	0.64
Smoking status (adults ≥20 year)				
Current	866	17.1	56.2 (52.9, 59.5)	57.4 (54.5, 60.4)
Former	1161	24.8	57.3 (54.4, 60.1)	55.4 (52.8, 58.0)
Never [ref]	2714	58.0	55.0 (52.6, 57.4)	55.1 (52.8, 57.5)
Current supplement use (adults ≥20 year)				
None [ref]	2047	41.8	54.6 (52.5, 56.7)	56.0 (54.0, 58.0)
T1:1–2 products/past 30 days	1660	33.1	55.5 (52.6, 58.5)	54.9 (52.2, 57.6)
T2:3 products/past 30 days	412	9.4	55.8 (52.0, 59.6)	54.1 (50.0, 58.2)
T3: ≥ 4 products/past 30 days	618	15.6	59.7 (56.3, 63.0) *	55.8 (52.8, 58.8)
*p*-trend			0.015	0.91

AFH stands for away-from home. Moderate-equivalent physical activity is calculated by multiplying the weekly duration of vigorous activities by 2 (e.g., 60 min of vigorous activity + 120 min of moderate activity week = 240 moderate-equivalent activity minutes). Asterisks indicate results of pairwise test comparing each proportion to the specified reference group identified as [ref] above: *** *p*-value < 0.001; ** 0.001 < *p*-value <0.01; * 0.01 < *p*-value < 0.05. Age-adjusted analyses not conducted for age group and for analyses limited to children.

**Table 3:** Proportion of population’s dietary energy (%E) impacted by FOPL among US adults (age ≥ 20 year) by health behaviors and conditions, 2017–2018.

	Mean Percent %E Impacted by FOPL (95% CI)	
	Crude	Age-adjusted
Body mass index, kg/m^2^		
Healthy weight (ref)	58.1 (54.5, 61.8)	58.9 (55.9, 61.8)
Overweight	56.2 (53.8, 58.7)	55.0 (52.6, 57.5) *
Obese	54.4 (51.8, 57.0) *	54.5 (52.0, 57.0) *
*p*-trend	0.036	0.009
Tried to lose weight in prior year		
No [ref]	57.7 (55.1, 60.2)	57.3 (54.8, 59.8)
Yes	54.2 (51.8, 56.7) *	53.9 (51.8, 55.9) *
Self-reported health status		
Excellent [ref]	58.5 (54.1, 62.9)	58.4 (54.4, 62.4)
Very good	53.7 (50.9, 56.4) *	53.6 (51.4, 55.8) *
Good	55.4 (52.4, 58.3)	55.4 (52.5, 58.4)
Fair/poor	59.1 (56.8, 61.3)	58.6 (56.2, 60.9)
*p*-trend	0.21	0.19
Diagnosed diabetes		
No [ref]	55.4 (53.2, 57.7)	55.7 (53.7, 57.7)
Yes	59.1 (56.6, 61.7) *	58.5 (53.8, 63.1)
Diagnosed hypertension		
No [ref]	55.5 (52.9, 58.0)	56.6 (54.4, 58.8)
Yes	56.5 (53.3, 59.7)	52.8 (48.9, 56.8)
Diagnosed high-cholesterol		
No [ref]	54.8 (52.6, 57.1)	56.0 (53.9, 58.1)
Yes	57.8 (54.8, 60.8)	53.5 (49.8, 57.3)
History of cardiovascular disease		
No [ref]	55.1 (53.1, 57.2)	55.4 (53.5, 57.3)
Yes	62.8 (57.7, 67.8) **	55.6 (47.4, 63.4)

Asterisk indicate *p*-value from pairwise comparison to specified reference group identified as [ref] above: ** 0.001 < *p* < 0.01; * 0.01 < *p* < 0.05.

**Table 4:** Proportion of population’s dietary constituents impacted by FOPL in the US, 2017–2018.

	Mean (SE)				
	Total Diet	FOPL	Not FOPL ^a^	Impacted by FOPL (95% CI)	*p*-Value of % Compared to Energy
Calories, kcal/d	2093 (14)	1186 (20)	907 (22)	56.6 (54.6, 58.7)	-
Macronutrients					
Total fat, g/d	85 (0.7)	48.3 (0.7)	36.8 (1)	56.8 (54.6, 58.9)	0.92
SFA, g/d	28 (0.3)	16.1 (0.3)	11.9 (0.3)	57.5 (55.2, 59.8)	0.43
MUFA, g/d	28.9 (0.3)	15.9 (0.2)	12.9 (0.4)	55.3 (53.1, 57.3)	0.17
PUFA, g/d	19.9 (0.3)	11.6 (0.3)	8.3 (0.2)	58.1 (55.9, 60.4)	0.19
Solid fats, g/d	36.2 (0.6)	22.3 (0.6)	13.9 (0.4)	61.7 (59.2, 64.1)	<0.001
Protein, g/d	78.3 (0.9)	34.8 (0.9)	43.4 (1.2)	44.5 (42.0, 47.0)	<0.001
Carbohydrate, g/d	246.9 (2.2)	157.5 (2.9)	89.4 (2.3)	63.8 (61.8, 65.8)	<0.001
Total sugars, g/d	107.9 (1.4)	74.9 (1.6)	33 (0.9)	69.4 (67.5, 71.4)	<0.001
Added sugars, tsp eq/d	16.9 (0.4)	13.2 (0.3)	3.8 (0.2)	77.8 (75.6, 80.0)	<0.001
Selected micronutrients					
Sodium, mg/d	3390 (34.7)	1587 (29.6)	1803 (44)	46.8 (44.8, 48.9)	<0.001
Fiber, g/d	16.2 (0.3)	8.7 (0.2)	7.5 (0.2)	53.9 (52.1, 55.7)	0.005
Potassium, mg/d	2497 (31)	1102 (20.3)	1395 (27.5)	44.1 (42.6, 45.7)	<0.001
Calcium, mg/d	968 (13)	627(13.9)	341 (8.5)	64.7 (62.8, 66.6)	<0.001
Vitamin D, μg/d	4.3 (0.1)	2.5 (0.1)	1.8 (0.1)	58.7 (55.8, 61.5)	0.15
Iron, mg/d	14 (0.2)	8.2 (0.2)	5.7 (0.2)	58.9 (56.4, 61.4)	0.071
Food groups					
Total fruit, cups/d	0.93 (0.04)	0.28 (0.02)	0.65 (0.03)	29.8 (26.5, 33.2)	<0.001
Whole fruit, cups/d	0.7 (0.03)	0.08 (0.01)	0.62 (0.03)	11.5 (9.7, 13.3)	<0.001
Fruit juice, cups/d	0.23 (0.01)	0.2 (0.01)	0.04 (0.01)	84.4 (81.3, 87.4)	<0.001
Total vegetables, cups/d	1.39 (0.04)	0.37 (0.01)	1.02 (0.04)	26.5 (24.3, 28.8)	<0.001
Dark-green vegetables, cups/d	0.14 (0.01)	0.02 (0.01)	0.12 (0.01)	16.9 (8.0, 25.7)	<0.001
Red/orange vegetables, cups/d	0.35 (0.01)	0.13 (0.01)	0.22 (0.01)	36.5 (32.7, 40.3)	<0.001
Tomato, cups/d	0.26 (0.01)	0.11 (0.01)	0.15 (0.01)	42.2 (37.7, 46.6)	<0.001
Potatoes, cups/d	0.36 (0.01)	0.12 (0.01)	0.24 (0.01)	33.1 (29.7, 36.5)	<0.001
Other starchy, cups/d	0.06 (0.01)	0.03 (0.01)	0.03 (0.01)	49.9 (42.5, 57.4)	0.074
Other vegetables, cups/d	0.48 (0.02)	0.07 (0.01)	0.41 (0.02)	13.9 (11.2, 16.6)	<0.001
Total dairy, cups/d	1.53 (0.03)	1.07 (0.03)	0.47 (0.01)	69.6 (67.3, 72.0)	<0.001
Milk, cups/d	0.69 (0.02)	0.57 (0.02)	0.13 (0.01)	81.7 (79.0, 84.3)	<0.001
Cheese, cups/d	0.74 (0.02)	0.42 (0.02)	0.33 (0.01)	56.1 (52.7, 59.4)	0.73
Yogurt, cups/d	0.06 (0.01)	0.06 (0.01)	0.01 (0.0)	91.6 (87.8, 95.3)	<0.001
Protein foods					
Meat, poultry & seafood, oz/d	4.5 (0.1)	1.1 (0.1)	3.3 (0.1)	25.6 (22.7, 28.4)	<0.001
Unprocessed red meat, oz/d	1.45 (0.08)	0.16 (0.02)	1.29 (0.08)	11.2 (8.3, 14.1)	<0.001
Processed meat, oz/d	0.92 (0.04)	0.61 (0.03)	0.31 (0.02)	66.5 (63.3, 69.8)	<0.001
Poultry, oz/d	1.56 (0.07)	0.2 (0.02)	1.35 (0.06)	13.0 (11.1, 14.9)	<0.001
Seafood, oz/d	0.53 (0.04)	0.17 (0.03)	0.36 (0.03)	31.3 (20.9, 41.8)	<0.001
Organ meat, oz/d	0.01 (0.01)	0.00 (0.00)	0.01 (0.01)	2.1 (0.0, 6.2)	<0.001
Legumes, cups/d	0.11 (0.01)	0.08 (0.01)	0.03 (0.01)	72.5 (67.5, 77.5)	<0.001
Nuts/seeds, oz/d	0.72 (0.05)	0.68 (0.05)	0.05 (0.01)	93.6 (92.6, 94.7)	<0.001
Soy, oz/d	0.09 (0.01)	0.05 (0.01)	0.03 (0.01)	61.8 (49.5, 74.1)	0.39
Eggs, oz/d	0.57 (0.02)	0.05 (0.01)	0.52 (0.02)	8.4 (7.3, 9.6)	<0.001
Total grains, oz/d	6.64 (0.11)	4.32 (0.13)	2.32 (0.1)	65.1 (61.9, 68.2)	<0.001
Refined grains, oz/d	5.8 (0.09)	3.58 (0.1)	2.22 (0.09)	61.7 (58.5, 64.9)	0.004
Whole grains, oz/d	0.84 (0.05)	0.74 (0.04)	0.10 (0.01)	88.3 (85.6, 91.1)	<0.001

^a^ Includes away-from-home foods and foods from stores that are not subject to FOPL. SE stands for standard error. SFA stands for saturated fatty acids, MUFA stands for monounsaturated fatty acids and PUFA stands for polyunsaturated fatty acids.

## Data Availability

The data used here are publicly available on the NHANES (https://wwwn.cdc.gov/nchs/nhanes/continuousnhanes/default.aspx?BeginYear=2017, accessed on 8 June 2022) and USDA websites (https://www.ars.usda.gov/northeast-area/beltsville-md-bhnrc/beltsville-human-nutrition-research-center/food-surveys-research-group/docs/fped-overview/, accessed on 8 June 2022).
